# Critical Literacy for Information Professionals

**DOI:** 10.5195/jmla.2017.115

**Published:** 2017-01

**Authors:** Mary A. Wickline

Critical literacy is an important and highly relevant concept to hospital and health sciences librarians: the theory reflects the value our profession gives to informed, contextual, critical readings of the research literature; the importance of context in evidence-based practice; and the inclusion and consideration of the patient’s and family’s social or cultural needs, values, and preferences. To wit, “the approach taken in critical literacy is not to read texts in isolation, but to develop an understanding of the cultural, ideological and sociolinguistic contexts in which they are created and read” (p. xi).

Sarah McNicol has gathered the work of twelve international authors from a variety of institutions—public libraries, school libraries, four-year college or university libraries, and a private college—to discuss various aspects of critical literacy as it relates to librarianship. Among the librarian-authors, only one is a health sciences librarian. The book has seven theoretical chapters and seven practical case studies, and each individual chapter is referenced. In the back of the book are an index and bibliographies for further reading on critical pedagogy, critical literacy, libraries and critical literacy, teaching resources, practical resources to support the teaching of critical literacy using comics, and new literacies and new literacy studies.

The greatest value for medical librarians is likely McNicol’s introduction and some of the theoretical chapters. McNicol presents various definitions of critical literacy and makes clear that it is as much about power and position as about the functional cognitive ability to read critically. She admonishes readers in her first chapter to remember that “all texts are constructed and serve particular interests” (p. xi).

It may feel, to some readers of this journal, that “critical literacy” is but an extension of “information literacy.” This is not entirely wrong, but it is a limited view. Jessica Critten’s theoretical chapter relates critical literacy to information literacy and delves deeper into the discipline of critical theory. Human reverence for the written word requires that we remind ourselves to critically consider the authority of the author against the life experience and perspective of the reader and, in medicine, to find the balance between well-designed, rigorous studies and the specific context of a particular patient.

A theoretical chapter on “Reading Health-Education Comics Critically: Challenging Power Relationships” is one example of “new literacies,” reminding readers that the format or delivery of information can affect its reception and clarity, while pointing out that comics can represent both hemispheres of our brains, conveying emotions along with the information contained in the accompanying words. McNicol differentiates the common use of “health literacy” as a simple focus on delivery of information versus the more critical consideration that includes social determinants of health.

The chapter then reports on a qualitative study about the use of comics and offers many quotes from participants. The chapter on “Using New Literacies to Discuss Disability in the Library” instructs readers to guard against an “abler…medical model” of disability limited to Americans with Disabilities Act (ADA) compliance and to aim for an “include” mindset with a universal design approach that works better for everyone (pp. 60–3). The author notes that not all disabilities are physical and that the ADA Amendments Act of 2008 (ADAAA) expanded consideration to conditions such as post-traumatic stress disorder (PTSD), autism, dementia, and other less visible disabilities. This author recommends various social broadcasting tools to gather and understand employee attitudes about accessibility as well as to gather patron expertise for improvements. This chapter, though, feels misplaced as a theoretical chapter: it should be in part 2 because its strength is in its practical examples and suggestions.

The writing in this book is uneven. Some writers understand the topic deeply (Critten, McNicol) and write incisively, while others slog through as if reproducing a dissertation: “here’s what I’ll tell you; I tell it; here’s what I told you.” There is definite value in the content—eventually, but an introductory paragraph should lead readers to why they should care, or the gap in what they know, or here is an exciting thing they can change, instead of defeating the intention with negativity before even beginning a topic. Then there are the run-on sentences that are difficult to follow. Some writers need editing, which unfortunately was not provided by the compilers of this anthology.

Education studies programs, linguistics departments, and communication departments will be familiar with critical theory as the extension of the work of Paolo Freire [1] or Jean Anyon [2], among others. While librarians may be familiar with the Framework for Information Literacy, we can too easily fall into the day-to-day practical work of “finding the answer” to an imposed query or an assigned question from busy doctors or nurses who are seeking quick, specific patient-related information (or the unfortunate “can you find me articles that support…”). We like to presume clinicians will bring their expertise and critical minds to whatever we find and offer. This book does us the service of expanding our knowledge of forms, formats, and depths of thinking related to multiple literacies. It is an introductory anthology that can help us deepen our consciousness of critical literacy.

McNicol is a doctoral research associate at the Education and Social Research Institute of Manchester Metropolitan University. The book is published by the Chartered Institute of Library and Information Professionals (CILIP), based in London. Their laudable goal is to put information and library skills and professional values at the heart of a democratic, equal, and prosperous society.

*Critical Literacy for Information Professionals* is useful and achieves what it sets out to do: offer a broad introduction to the theory of critical literacy, with practical examples that could be transferable to other settings. The practical chapters heavily emphasize primary and secondary school or undergraduate programs, but none specific to hospital or medical libraries as the two chapters directly related to health sciences are theoretical chapters. However, much of this work could still be adaptable, by any thoughtful librarian, to a medical or hospital context. Trudi Jacobson and Thomas Mackey’s books on meta-literacy [3, 4] might offer a more in-depth approach to the topic.
